# Common Variants in Osteopontin and *CD44* Genes as Predictors of Treatment Outcome in Radiotherapy and Chemoradiotherapy for Non-Small Cell Lung Cancer

**DOI:** 10.3390/cells12232721

**Published:** 2023-11-28

**Authors:** Seweryn Gałecki, Agnieszka Gdowicz-Kłosok, Regina Deja, Barbara Masłyk, Monika Giglok, Rafał Suwiński, Dorota Butkiewicz

**Affiliations:** 1 Center for Translational Research and Molecular Biology of Cancer, Maria Skłodowska-Curie National Research Institute of Oncology, Gliwice Branch, 44-102 Gliwice, Poland; 2 Department of Systems Biology and Engineering, Silesian University of Technology, 44-100 Gliwice, Poland; 3 Analytics and Clinical Biochemistry Department, Maria Skłodowska-Curie National Research Institute of Oncology, Gliwice Branch, 44-102 Gliwice, Poland; 4 II Radiotherapy and Chemotherapy Clinic and Teaching Hospital, Maria Skłodowska-Curie National Research Institute of Oncology, Gliwice Branch, 44-102 Gliwice, Poland

**Keywords:** osteopontin, OPN, SPP1, CD44, lung cancer, polymorphism, cancer progression, metastasis, prognosis, radiotherapy, curative treatment

## Abstract

Osteopontin (OPN)-CD44 signaling plays an important role in promoting tumor progression and metastasis. In cancer, OPN and CD44 overexpression is a marker of aggressive disease and poor prognosis, and correlates with therapy resistance. In this study, we aimed to evaluate the association of single nucleotide polymorphisms (SNPs) in the *OPN* and *CD44* genes with clinical outcomes in 307 non-small cell lung cancer (NSCLC) patients treated with radiotherapy or chemoradiotherapy. The potential impact of the variants on plasma OPN levels was also investigated. Multivariate analysis showed that *OPN* rs11730582 CC carriers had a significantly increased risk of death (*p* = 0.029), while the *CD44* rs187116 A allele correlated with a reduced risk of locoregional recurrence (*p* = 0.016) in the curative treatment subset. The rs11730582/rs187116 combination was associated with an elevated risk of metastasis in these patients (*p* = 0.016). Furthermore, the *OPN* rs1126772 G variant alone (*p* = 0.018) and in combination with rs11730582 CC (*p* = 7 × 10^−5^) was associated with poor overall survival (OS) in the squamous cell carcinoma subgroup. The rs11730582 CC, rs187116 GG, and rs1126772 G, as well as their respective combinations, were independent risk factors for unfavorable treatment outcomes. The impact of rs11730582-rs1126772 haplotypes on OS was also observed. These data suggest that *OPN* and *CD44* germline variants may predict treatment effects in NSCLC.

## 1. Introduction

Lung cancer continues to be the leading cause of cancer deaths worldwide [1]. Despite the introduction of targeted therapies and immunotherapy, radiotherapy and platinum-based chemoradiotherapy are still the mainstay of treatment in locally advanced and inoperable non-small cell lung cancer (NSCLC). However, common drug and radiation resistance impacts the effectiveness of these treatments and contributes to progression and poor prognosis [2]. At the same time, conventional clinical factors used to guide therapeutic decisions are not able to precisely predict the patients’ outcome. Hence, it is necessary to search for factors that may help in the assessment of treatment effects and prognosis in NSCLC in order to identify risk groups and select an optimized therapeutic strategy.

Osteopontin (OPN), also known as secreted phosphoprotein 1 (SPP1), is a multifunctional glycoprotein and extracellular matrix (ECM) component that mediates a variety of physiological and pathological processes. It is involved in tumorigenesis and metastasis, including cell proliferation, adhesion, invasion, migration, angiogenesis, apoptosis, autophagy, and immune response [3]. In many solid tumors, including lung cancer, OPN overexpression in the tumor and increased circulating levels are markers of an aggressive phenotype and/or unfavorable prognosis [3,4]. For example, high OPN levels correlated with tumor growth and lymphatic metastasis in several lung cancer studies [5,6], while OPN knockdown inhibited the invasion and metastasis of NSCLC cells [7]. OPN expression was also associated with reduced apoptotic activity in lung adenocarcinoma [8]. In NSCLC patients, a relationship was found between increased levels of OPN in the tumor and serum/plasma and advanced disease, poor treatment response, and survival outcomes [9,10,11,12]. In our previous study, high pretreatment plasma OPN levels were significantly associated with unfavorable survival in inoperable NSCLC, especially in patients with squamous cell carcinoma [13]. Moreover, OPN expression may correlate with hypoxia and mediate resistance to radiotherapy and cytotoxic drugs [14]. High pretreatment OPN levels were related to the poor oxygenation status of NSCLC patients treated with curative-intent radiotherapy [12]. Blocking the OPN gene in combination with irradiation led to the decreased viability of breast cancer cells and induction of apoptosis, which highlights the role of OPN in the response to ionizing radiation [15]. In glioblastoma, OPN inhibition resulted in increased radiosensitivity and tumor size reduction in vivo [16]. It was found that autophagy-induced OPN suppression abrogated the radioresistance of NSCLC cells [17]. OPN has also been shown to promote cisplatin resistance in small cell lung cancer cells, mainly by inhibiting apoptosis [18]. In NSCLC, OPN expression significantly correlated with distant metastasis and response to platinum-based chemotherapy [19].

OPN promotes tumor progression through binding to CD44 and integrin cell receptors. CD44 is a transmembrane cell surface glycoprotein and a marker for cancer stem cells in many solid tumors [20]. Data show that OPN-CD44 signaling is an important factor influencing cancer aggressiveness [3,21,22]. In addition to OPN, CD44 ligands also include hyaluronic acid, matrix metalloproteinases (MMPs), and growth factors. CD44 regulates proliferation, invasion, migration, and stemness, and its overexpression is associated with cancer recurrence and metastasis [23]. In NSCLC, high CD44 levels promoted cell proliferation and colony formation [24]. Primary lung tumors with highly expressed CD44 demonstrated increased metastasis to the regional lymph nodes, and CD44 enhanced the ability of lung cancer cells to migrate and invade [25]. CD44 overexpression may also contribute to drug and radiation resistance, as well as poor prognosis in various malignancies [22,26]. For example, CD44 knockdown was associated with enhanced chemo- and radiosensitivity and reduced epithelial-mesenchymal transition in prostate cancer cells [27]. The CD44(+) gastric cancer cells exhibited increased resistance to chemotherapy- or radiation-induced cell death [28]. In the glioma model in vivo, CD44 promoted cancer stem cell phenotype and radiation resistance, while CD44 expression correlated with hypoxia-induced gene signatures and poor survival in glioblastoma patients [21]. In lung cancer, CD44 downregulation was involved in sensitization to cisplatin and gefitinib, whereas lower CD44 expression in tumors was associated with better recurrence-free survival [29]. It was also demonstrated that CD44 was upregulated in radiation-survived NSCLC cells which could suggest its role as a marker of radiotherapy response in NSCLC [30].

Common germline genetic variants, such as single nucleotide polymorphisms (SNPs), especially in the promoter and regulatory regions, may modulate protein levels and activity, consequently affecting therapy results and disease progression in lung cancer. Most research on the prognostic role of OPN and CD44 in cancer focuses on protein expression levels. The data in the literature on the *OPN* and *CD44* SNPs and clinical outcomes in solid tumors usually refer to Asian populations and the results are inconclusive [31]. Moreover, there are very few such studies in lung cancer. Therefore, in this report we aimed to evaluate the association between common SNPs in the *OPN* (also known as *SPP1*) and *CD44* genes and three survival endpoints, as well as the potential relationship with circulating OPN levels before treatment, in patients with inoperable NSCLC receiving radiotherapy (RT) alone or in combination with chemotherapy (CTRT). To our knowledge, this is the first study of this type conducted in Caucasian NSCLC patients. Some of the analyzed variants (e.g., *OPN* rs1126772 or *CD44* rs187116) have never been investigated in lung cancer before.

## 2. Materials and Methods

### 2.1. Study Population

A group of 307 Caucasian patients with inoperable NSCLC was treated and recruited at the Maria Skłodowska-Curie National Research Institute of Oncology in Gliwice. The mean age at diagnosis was 64.0 years (median, 64; range 33–84 years). Most of the cases were at advanced clinical stage III or IV (90%), had a Zubrod performance status (PS) of 0–1 (91%) and had a history of cigarette smoking (94%). Squamous cell carcinoma (SCC) was diagnosed in 181 (59%) patients, adenocarcinoma (AC) was diagnosed in 51 (17%) patients, and 75 (24%) patients had NSCLC not otherwise specified (NOS). Patient characteristics are shown in Table 1. All patients received RT with a total dose ≥ 20 Gy, and 216 (70%) patients were given platinum-based CT. Out of all patients, 145 (47%) individuals with stage I–III were qualified to treatment with curative intent, i.e., thoracic RT at a total dose ≥ 60 Gy (range 60–72 Gy). Induction CT (i.e., 2–4 courses) was administered to 92% of these patients. The treatment details have been described in our previous study [13].

### 2.2. SNP Identification

Five common SNPs were examined in this study, including *OPN* rs1126772, *OPN* rs11730582, *OPN* rs4754, *CD44* rs187116, and *CD44* rs13347. The following selection criteria were used: variants had a minor allele frequency (MAF) in the European Caucasian population ≥ 20% [32], were associated with cancer and located in regulatory or coding regions or in domains relevant to protein activity, and/or had potential or documented functional significance (Table S1) [33,34,35,36,37].

Genomic DNA was extracted from frozen peripheral blood collected during routine diagnostic tests prior to treatment. The rs1126772, rs4754, and rs187116 SNPs were identified with polymerase chain reaction-restriction fragment length polymorphism (PCR-RFLP) method. The primers used for PCR are shown in Table S2. The reaction was performed in 25 µL of total volume containing 50 ng of genomic DNA, 0.2 mM of each dNTP, 12.5 pmol of each primer (Genomed, Warszawa, Poland), 1× PCR buffer, 1.5 mM MgCl_2_, 0.5 U of Perpetual Taq DNA polymerase (Eurx, Gdańsk, Poland). The initial denaturation at 95 °C for 5 min was followed by 35 cycles of denaturation at 95 °C for 30 s, annealing at 57 °C (for rs1126772) or 55 °C (rs4754 and rs187116) for 30 s and elongation at 72 °C for 30 s, ending with 72 °C for 5 min. Then, PCR products were digested overnight with 5 U BfaI (for rs1126772), or BbsI (for rs4754), or MspI (for rs187116) restriction enzymes (New England Biolabs, Ipswich, MA, USA), and the fragments were separated on 3–4% ethidium bromide-stained agarose gels. The rs11730582 and rs13347 genotypes were determined using C_1840808_20 and C_7619022_10 TaqMan SNP Genotyping Assays (Applied Biosystems, Foster City, CA, USA), respectively, according to the manufacturer’s standard protocol. Genotyping was repeated in 30 randomly selected samples and 100% concordance was found.

### 2.3. Measurement of Plasma OPN

Blood samples were collected before treatment and processed as previously described [13]. Plasma samples were stored at −80 °C until analysis. Circulating OPN levels were measured using an enzyme-linked immunosorbent assay (ELISA) using Human OPN Quantikine ELISA kit, DOST00 (R&D Systems Inc., Minneapolis, MN, USA), according to the manufacturer’s instructions.

### 2.4. Statistical Analysis

Clinical endpoints of the study included overall survival (OS), locoregional recurrence-free survival (LRFS), and metastasis-free survival (MFS). OS was calculated from diagnosis until the date of death or last known date alive, while LRFS and MFS were calculated from the date of treatment initiation to the date of documented locoregional progression (for LRFS) or the date of distant relapse (for MFS), or last follow-up evaluation. Survival curves were determined with the Kaplan–Meier method and compared with the log-rank test. The SNPs were tested under dominant, recessive, and additive genetic models, and the model with the most significant *p* value was selected for further analysis. The hazard ratios (HRs) with 95% confidence intervals (CIs) were estimated using univariate and multivariate Cox proportional hazard regression models. Multivariate models were adjusted for median age at diagnosis (<64 versus ≥64 years), sex (male versus female), histology (SCC versus non-SCC), clinical stage (I–II versus III versus IV), Zubrod PS (0–1 versus 2), smoking (ever versus never), CT use (yes versus no), and RT dose (<60 versus ≥60 Gy). A backward stepwise multiple regression was performed to identify independent risk factors. Haplotypes and their frequencies were estimated using PHASE v2.1.1 [38]. Haplotype blocks with *D*′ and *r*^2^ parameters used to estimate the degree of linkage disequilibrium (LD) between SNPs were determined in the Haploview v4.2 software [39,40] according to Garbiel et al. [41]. The Kruskall–Wallis H test and the Mann–Whitney U test were used to compare OPN levels between groups. Spearman’s correlation and Pearson’s chi-square test were applied to evaluate the associations between variables. All tests were two-tailed and the *p* value was considered significant at 0.05. Statistical analyses were performed using Statistica 13.3 (TIBCO Software Inc., Palo Alto, CA, USA) and R v3.6.1 software (The R Foundation for Statistical Computing, Vienna, Austria, https://www.r-project.org, accessed on 4 December 2022).

## 3. Results

The median OS in the group was 18.3 months, the median LRFS was 18.6 months, and the median MFS was 28.7 months. The median follow-up time was 40.9 months. During observation time 218 (71%) deaths occurred, while 102 (33%) patients experienced locoregional recurrence, and 78 (25%) patients developed distant metastasis. The analysis showed that age ≥ 64 years (*p* = 0.011), male sex (*p* = 0.032), SCC histology (*p* = 0.013), clinical stage IV (*p* = 0.024), PS 2 (*p* = 0.016), smoking (*p* = 0.001), lack of CT (*p* = 1 × 10^−6^), and RT dose < 60 Gy (*p* < 1 × 10^−6^) correlated with poor OS, whereas SCC histology (*p* = 0.017), clinical stage IV (*p* = 0.052), PS 2 (*p* = 0.005), smoking (*p* = 0.041), lack of CT (*p* = 4 × 10^−5^), and RT dose < 60 Gy (*p* = 2 × 10^−5^), as well as clinical stage IV (*p* < 1 × 10^−6^) and RT dose < 60 Gy (*p* < 1 × 10^−6^), were associated with unfavorable LRFS and MFS, respectively. There was no statistically significant relationship between the genotypes and clinico-demographic parameters, except that rs13347 correlated with use of CT (*p* = 0.035). The MAFs in the group were consistent with those found in the European Caucasian population [32], and the genotype distribution was in accordance with the Hardy–Weinberg law (Table S1).

### 3.1. OPN and CD44 SNPs and Survival Outcomes

There was no statistically significant effect of the studied SNPs on the outcome observed in the whole group. Only patients with the *CD44* rs187116 A variant had better LRFS than GG homozygotes (*p* = 0.083) in univariate models, and the *OPN* rs11730582 CC carriers showed an elevated risk of distant relapse in the multivariate analysis (HR 1.63, *p* = 0.064), but the associations were not significant (Table 2).

However, when a more homogeneous subgroup of patients treated with curative RT doses (i.e., ≥60 Gy) was analyzed separately, two SNPs were found to be significantly associated with the studied clinical endpoints. Patients with at least one *CD44* rs187116 A allele had significantly better LRFS than GG homozygotes (median LRFS 30.4 versus 22.0 months, *p* = 0.039). The rs187116 A variant carriers also demonstrated better MFS compared to GG homozygotes, but the difference was not statistically significant (median MFS not reached versus 24.8 months, *p* = 0.063). In multivariate analysis adjusted for clinical and demographic parameters, the *OPN* rs11730582 CC carriers had a significantly increased risk of death compared to T variant carriers (HR 1.74, *p* = 0.029) (Table 2). The rs11730582 C variant was also associated with an over two-fold increase in the risk of metastasis in multivariate model, but this was not statistically significant (*p* = 0.068). The *CD44* rs187116 A allele had a significant protective effect with respect to risk of locoregional recurrence in univariate and multivariate Cox models (HR 0.55, *p* = 0.033 and HR 0.48, *p* = 0.016, respectively). The rs187116 A variant carriers also showed a reduced risk of metastasis as compared to GG homozygotes in univariate and multivariate models; however, the association was not statistically significant (*p* = 0.054 and 0.076, respectively). The final model demonstrated that the *OPN* rs11730582 CC genotype (*p* = 0.042) together with SCC histological subtype and smoking were independent predictors of poor OS in the curative treatment subgroup (Table 3). In contrast, the *CD44* rs187116 A allele was an independent protective factor against locoregional recurrence (*p* = 0.016), whereas the SCC histology and advanced clinical stage were independent indicators of unfavorable LRFS.

Furthermore, as hypoxia is a much greater clinical problem in SCC than in AC and the OPN levels had strong prognostic value among SCC patients in our previous study [13], we assessed the effect of the studied SNPs in this subgroup. The rs11730582 CC homozygotes with SCC had shorter OS than T variant carriers (median OS 10.0 versus 19.3 months, *p* = 0.027). The CC genotype was also significantly associated with increased risk of death in the univariate model (HR 1.60, *p* = 0.015), but not in the multivariate model (*p* = 0.093) (Table 2). Patients with the *OPN* rs1126772 G allele demonstrated unfavorable OS compared to AA homozygotes (median OS 12.5 versus 17.8 months, *p* = 0.034). The G variant carriers were at significantly increased risk of death in both univariate and multivariate analyses (HR 1.54, *p* = 0.020 and HR 1.59, *p* = 0.018, respectively). The rs11730582 CC and rs1126772 G were also non-significantly associated with an increased risk of recurrence in univariate models (*p* = 0.096 and 0.095, respectively). In the final model, only the rs1126772 G variant was an independent risk factor for poor OS, together with smoking and an RT dose lower than 60 Gy (Table 3).

### 3.2. Cumulative Analysis

**Figure 1:**
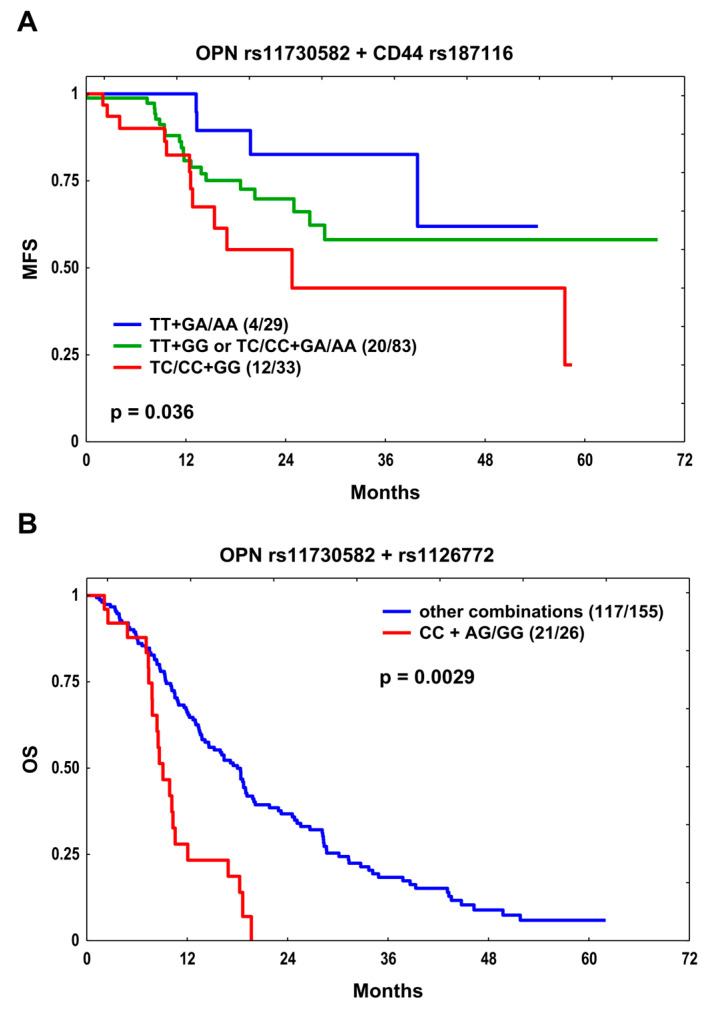
The Kaplan–Meier plots for (**A**) metastasis-free survival (MFS) with respect to the *OPN* rs11730582 and *CD44* rs187116 SNP combination in the curative treatment subgroup and for (**B**) overall survival (OS) with respect to the *OPN* rs11730582 and rs1126772 SNP combination in the squamous cell carcinoma (SCC) subgroup. Number of events and *n* are shown in the brackets.

In order to examine whether the co-occurrence of SNPs had a stronger effect on clinical outcomes than single variants, we constructed for each endpoint the genotype combinations for SNPs with *p* ≤ 0.100 in the multivariate analysis (Table 2). Thus, there were two combinations meeting this criterion. One of them was possible for MFS in the curative treatment subgroup and involved *OPN* rs11730582 and *CD44* rs187116. The unfavorable genotypes were TC/CC and GG, respectively. MFS was significantly shorter in carriers of both adverse genotypes compared to TT + GA/AA carriers (median MFS 24.8 months versus median not reached, *p* = 0.036; Figure 1A); however, no interaction was observed (likelihood ratio test *p* = 0.123). Patients carrying the unfavorable rs11730582 TC/CC + rs187116 GG combination had a 3.6-fold and more than 4-fold higher risk of developing metastasis compared to TT + GA/AA carriers in univariate (HR 3.59, *p* = 0.028) and multivariate models (HR 4.19, *p* = 0.016; Table 2), respectively. In the final model, this SNP combination was the only independent risk factor for metastasis in patients treated with curative intent (HR 2.05, *p* = 0.043) (Table 3). The second combination was possible for OS in the SCC subgroup and involved *OPN* rs11730582 and rs1126772. The adverse genotypes were CC and AG/GG, respectively. Patients with two risk genotypes showed significantly reduced OS compared to non-carriers (median OS 9.1 versus 18.0 months, *p* = 0.0029; Figure 1B) and an interaction was found between these SNPs (rs11730582 versus rs11730582/rs1126772, *p* = 0.013 and rs1126772 versus rs11730582/rs1126772, *p* = 0.003; likelihood ratio test *p* = 0.0024). The presence of the rs11730582 CC + rs1126772 AG/GG combination conferred nearly a three-fold increase in risk of death in univariate (HR 2.82, *p* = 3.3 × 10^−5^) and multivariate analyses (HR 2.74, *p* = 7 × 10^−5^; Table 2). The final model showed that CC + AG/GG combination was strongly and independently associated with unfavorable OS in the SCC subgroup, together with smoking and RT dose below 60 Gy (Table 3).

### 3.3. OPN Haplotypes and Clinical Outcome

The influence of haplotypes on survival outcomes was assessed only for SNPs exhibiting strong LD, i.e., for *OPN* rs11730582-rs1126772 (*D*′ = 1.0, 95% CI 0.96–1.0) and rs4754-rs1126772 (*D*′ = 0.96, 95% CI 0.86–0.99) (Figure S1). The rs11730582-rs1126772 haplotype frequencies in the group were: 52.5% for T-A, 28.8% for C-A, 18.2% for C-G, and 0.5% for T-G. The rs4754-rs1126772 haplotype frequencies were: 75.1% for T-A, 18.7% for C-G, 6.2% for C-A, and 0% for T-G. The analysis was conducted only for haplotypes more frequent than 1%. There was no statistically significant relationship between haplotypes and clinical outcome in the whole patient group. In the curative treatment subset, patients carrying at least one rs4754-rs1126772 C-A copy had better OS (median OS 37.5 versus 21.6 months, *p* = 0.045) and non-significantly longer LRFS (median LRFS 38.0 versus 20.3 months, *p* = 0.070) compared to non-carriers (Figure S2A,B); however, this was not confirmed in the multivariate analysis (*p* = 0.123 and 0.312, respectively). The presence of at least one rs11730582-rs1126772 C-A copy was significantly associated with increased risk of death in the multivariate model (HR 1.81, *p* = 0.007) (Table 4). Moreover, the C-A haplotype carriers showed non-significantly reduced MFS (*p* = 0.077, Figure S2C) as well as an elevated risk of metastasis in univariate (HR 1.81, *p* = 0.082) and multivariate models (HR 2.02, *p* = 0.053) when compared to non-carriers. In the final model, the rs11730582-rs1126772 C-A haplotype was an independent indicator of poor OS (HR 1.70, 95% CI 1.11–2.59, *p* = 0.014), together with age ≥ 64 years, SCC histology, and smoking. In the SCC subgroup, carriers of the rs4754-rs1126772 C-A haplotype had longer LRFS compared to non-carriers (median LRFS 31.5 and 15.2 months, *p* = 0.011; Figure S3A) and a significantly lower risk of recurrence in univariate model (HR 0.35, 95% CI 0.13–0.97, *p* = 0.044) but not in the multivariate model (*p* = 0.110). The rs11730582-rs1126772 T-A haplotype was associated with better OS (*p* = 0.026; Figure S3B) and significantly lower risk of death in the univariate model (HR 0.62, *p* = 0.014) but not in the multivariate analysis (*p* = 0.098) (Table 4). The SCC patients with rs11730582-rs1126772 C-G demonstrated shorter OS than non-carriers (median OS 12.1 versus 18.0 months, *p* = 0.033; Figure S3C). The C-G haplotype was also associated with significantly increased risk of death in both the univariate (HR 1.55, *p* = 0.019) and multivariate models (HR 1.61, *p* = 0.016; Table 4). The final model showed that the C-G haplotype was an independent indicator of unfavorable OS (HR 1.60, 95% CI 1.10–2.24, *p* = 0.018) in the SCC subgroup, together with smoking and RT dose < 60 Gy.

### 3.4. OPN Levels and SNPs

The mean ± standard deviation (SD) OPN levels were 119.5 ± 66.1 ng/mL (median 104.0, range 6.5–674.3) in the entire group, 106.1 ± 68.3 ng/mL (median 90.2, range 6.5–674.3) in the curative treatment subset, and 124.0 ± 72.0 (median 106.9, range 6.5–674.3) in the SCC subset. The prognostic value of plasma OPN concentration in relation to clinical parameters in this NSCLC cohort has been investigated in our previous report [13]. In the current study, there was no statistically significant association between *OPN* genotypes or haplotypes, as well as tested SNP combinations, and circulating OPN levels before treatment neither in all patients nor in both patient subsets examined (Table S3).

## 4. Discussion

In this report, using multivariate models, we demonstrated a statistically significant effect of the *OPN* rs11730582 and *CD44* rs187116 SNPs, as well as *OPN* rs11730582-rs1126772 haplotype on survival outcomes in inoperable NSCLC patients treated with curative intent. Our observation that the rs11730582 CC genotype was independently associated with decreased OS confirmed the results of two previous lung cancer studies in the Chinese population, in which Hao et al. [42] showed a correlation of the C variant with a worse response to platinum-based CT and poor prognosis in patients with inoperable stage IIIB-IV NSCLC, while Chen et al. [43] reported shorter survival and an increased incidence of bone metastases in CC homozygotes. In the only published study involving Caucasian patients, the authors found no association with prognosis, local recurrence, and metastasis in stage I–III NSCLC [9]. Similar to our data, CC homozygotes had significantly lower survival rates and higher susceptibility to gastric cancer [35,44], as well as increased invasiveness and risk of thyroid cancer [45]. However, in the case of other solid tumors, such as, e.g., glioma or oral, nasopharyngeal, hepatocellular, and breast cancers, variant C was protective in terms of cancer risk or prognosis [36,46,47,48,49]. The results of a meta-analysis based on 11 studies in the Chinese population, including the NSCLC study, suggested in turn that rs11730582 had no effect on cancer risk [50].

Consistent with the above-mentioned data, we also identified the C-A haplotype of the rs11730582 and rs1126772 as an independent indicator of poor OS in the curative treatment subset. Moreover, in our dataset, the *OPN* rs1126772 GG genotype and the rs11730582-rs1126772 C-G haplotype were independently associated with unfavorable OS in patients with SCC. In addition, we observed a strong independent effect of the rs11730582 CC and rs1126772 G combination on prognosis in this subgroup. The rs1126772 A>G SNP in the 3′ untranslated region (3′UTR) may deregulate the *OPN* gene expression and protein production. Although, to our knowledge, it has not been functionally tested, it was predicted to be within the miR-23a, miR-23b, and miR-371-5p binding sites [51], which might suggest its potential phenotypic effect. This SNP has been very rarely studied in cancer disease, with the only finding that the G variant was associated with an increased risk of gastric cancer [52]. In turn, the rs11730582 -443T>C functional polymorphism located within the gene promoter is one of the most frequently investigated *OPN* gene variants. It was found to cause a differential binding of unknown nuclear factor, which may be the MYT1 transcription factor [33]. The study by Schultz et al. in melanoma cells showed enhanced transcription for the -443C variant associated with allele-specific binding of c-Myb to the promoter region [34]. In gastric cancer, the C variant resulted in significantly higher promoter activity [35]. Elevated OPN protein levels were also observed in melanoma cell lines homozygous for C allele, as well as in tumor tissue from thyroid cancer patients with CC genotype [34,45]. However, in a single study, Dong et al. demonstrated the opposite relationship using a hepatocellular carcinoma model, namely, the T allele caused higher transcriptional activity and protein expression leading to a significant increase of tumor growth and metastasis [36]. These data suggest that rs11730582 T>C promoter SNP may be one of the factors modulating the OPN gene and protein expression levels. OPN overexpression is known to correlate with the aggressiveness and poor outcome in lung cancer [5,9,10,13,53]. Functional studies mostly indicate that variant C may confer a higher level of OPN, which is in line with the results of our study and other reports regarding lung, gastric, and thyroid cancers. However, it is likely that the effect direction of this SNP may be context-dependent. Based on the existing data, it can therefore be speculated that both rs11730582 and rs1126772 contribute to unfavorable prognosis in NSCLC as OPN upregulation promotes tumor progression and metastasis, as well as playing a role in resistance to anticancer drugs, including platinum agents and ionizing radiation [14].

Another interesting finding in our study was the protective effect of the *CD44* rs187116 A variant with respect to the risk of locoregional recurrence after curative treatment. Although the functional significance of this SNP is unknown, the rs187116 G>A substitution is located in intron 1 and this may affect the transcriptional activity of the gene and splicing regulation. In lung cancer, data on the role of various *CD44* SNPs are very limited, while the rs187116 has not been studied at all. Nevertheless, an effect of this SNP consistent with our observations has been previously reported for gastric cancer. For example, both Winder et al. [54] as well as Bitaraf et al. [55] showed that the unfavorable G allele was associated with poor OS and a higher risk of tumor recurrence in these patients. In a Japanese study, the G variant carriers had shorter disease-free survival compared to AA homozygotes [56]. Increased CD44 expression has also been demonstrated in gastric tissue in patients with the G variant [57]. Moreover, the G allele was found to correlate with a higher susceptibility to this type of cancer [55]. However, the opposite relationship has been reported for the risk of breast and colorectal cancer [58,59], whereas no association with prognosis and recurrence was found in a single study on colon carcinoma [60]. Interestingly, in our NSCLC group treated with curative intent, a joint effect of *OPN* rs11730582 and *CD44* rs187116 on the risk of developing metastases was also observed, while each of them individually showed no significant influence on MFS, which indicates the need to take multiple SNPs into account for risk stratification. This finding also highlights the possible usefulness of these variants in predicting lung cancer dissemination after radical therapy, especially since the OPN-CD44 axis is known to play a role in the progression of several solid tumors [21,22]. Both our results in NSCLC and those of other authors show that the *OPN* and *CD44* polymorphisms may be important modulators of the disease course and therapeutic response.

Finally, we found no association between the rs1126772, rs11730582, and rs4754 SNPs, as well as their haplotypes and pretreatment circulating OPN levels in our NSCLC cohort. Similar results were previously obtained by several other authors in lung cancer and melanoma patients [9,10,61]. However, rs11730582 in the promoter region was shown to be linked to serum/plasma OPN levels in nasopharyngeal and breast cancers [48,49]. These inconsistencies may reflect the complexity of the mechanisms influencing circulating OPN levels; thus, further research is required to elucidate the role of particular SNPs in OPN regulation.

## 5. Conclusions

In conclusion, this study identified for the first time three germline variants in the *OPN* and *CD44* genes that individually or jointly influenced clinical outcome and prognosis in inoperable NSCLC patients treated with curative RT doses and in patients with SCC. We demonstrated that rs187116, rs11730582, and rs1126772 were predictors of recurrence, metastasis, and poor survival independently of strong clinical and lifestyle factors. These findings may be of particular importance since SNPs can be easily detected in DNA from readily available peripheral blood samples prior to treatment. Our study, however, is subject to certain limitations, the largest of which includes a relatively small sample size and number of examined SNPs, as well as the so far poorly understood molecular mechanisms underlying the associations found. Therefore, our initial results should be verified in larger populations of cancer patients. If these observations were confirmed, certain *OPN* and *CD44* SNPs could become valuable additional information in predicting the response to RT and CTRT in NSCLC patients.

## Figures and Tables

**Table 1:** Characteristics of the patients.

Feature		All Patients *n* (%)	Curative Treatment *n* (%)
	Total	307 (100)	145 (100)
Age at diagnosis (median)	<64 years ≥64 years	146 (48) 161 (52)	84 (58) 61 (42)
Sex	Male Female	239 (78) 68 (22)	108 (74) 37 (26)
Histology NSCLC	SCC AC NOS	181 (59) 51 (17) 75 (24)	86 (59) 23 (16) 36 (25)
Clinical stage	I–II III IV	30 (10) 213 (69) 64 (21)	20 (14) 125 (86) 0 (0)
Performance status	0 1 2	81 (26) 199 (65) 27 (9)	57 (39) 88 (61) 0 (0)
Smoking status	Never smokers Ever smokers	19 (6) 288 (94)	13 (9) 132 (91)
Chemotherapy use	No Yes	91 (30) 216 (70)	12 (8) 133 (92)
Radiation dose	<60 Gy ≥60 Gy	162 (53) 145 (47)	- 145 (100)

NSCLC, non-small cell lung cancer; SCC, squamous cell carcinoma; AC, adenocarcinoma; NOS, not otherwise specified.

**Table 2:** Cox regression analysis in the whole group, in the curative treatment subset, and in patients with SCC (SNPs with *p* < 0.100 in uni- or multivariate models shown only).

Endpoint	SNP	Genotype	Events/*n*	*p* Log-Rank	HR (95% CI)	*p*	HR (95% CI) ^a^	*p*
All patients								
LRFS	*CD44* rs187116	GG GA/AA	42/105 60/202	0.084	1.0 0.70 (0.47–1.05)	0.083	1.0 0.73 (0.48–1.11)	0.139
MFS	*OPN* rs11730582	TT/TC CC	57/231 21/76	0.199	1.0 1.42 (0.86–2.34)	0.174	1.0 1.63 (0.97–2.74)	0.064
Curative treatment subgroup								
OS	*OPN* rs11730582	TT/TC CC	72/112 23/33	0.274	1.0 1.32 (0.82–2.11)	0.251	1.0 1.74 (1.06–2.86)	**0.029**
LRFS	*CD44* rs187116	GG GA/AA	22/48 32/97	**0.039**	1.0 0.55 (0.32–0.95)	**0.033**	1.0 0.48 (0.27–0.87)	**0.016**
MFS	*OPN* rs11730582	TT TC/CC	7/44 29/101	0.139	1.0 1.84 (0.80–4.21)	0.148	1.0 2.21 (0.94–5.16)	0.068
	*CD44* rs187116	GG GA/AA	15/48 21/97	0.063	1.0 0.52 (0.27–1.01)	0.054	1.0 0.53 (0.27–1.07)	0.076
MFS	*OPN* rs11730582 + *CD44* rs187116	TT + GA/AA TT + GG or TC/CC + GA/AA TC/CC + GG	4/29 20/83 12/33	**0.036**	1.0 2.04 (0.70–5.99) 3.59 (1.15–11.18)	0.193 **0.028**	1.0 2.36 (0.78–7.12) 4.19 (1.30–13.48)	0.126 **0.016**
SCC subgroup								
OS	*OPN* rs11730582	TT/TC CC	101/136 37/45	**0.027**	1.0 1.60 (1.09–2.34)	**0.015**	1.0 1.41 (0.94–2.11)	0.093
	*OPN* rs1126772	AA AG/GG	92/120 46/61	**0.034**	1.0 1.54 (1.07–2.22)	**0.020**	1.0 1.59 (1.08–2.33)	**0.018**
OS	*OPN* rs11730582 + rs1126772	Other combinations CC + AG/GG	117/155 21/26	**0.0029**	1.0 2.82 (1.73–4.60)	**3.3 × 10^−5^**	1.0 2.74 (1.67–4.51)	**7 × 10^−5^**
LRFS	*OPN* rs11730582	TT/TC CC	48/136 17/45	0.131	1.0 1.61 (0.92–2.80)	0.096	1.0 1.48 (0.82–2.66)	0.192
	*OPN* rs1126772	AA AG/GG	44/120 21/61	0.133	1.0 1.57 (0.92–2.67)	0.095	1.0 1.35 (0.75–2.42)	0.318

SNP, single nucleotide polymorphism; OS, overall survival; LRFS, locoregional recurrence-free survival; MFS, metastasis-free survival; HR, hazard ratio; CI, confidence interval; SCC, squamous cell carcinoma. ^a^ Adjusted for age at diagnosis, sex, smoking, clinical stage, histology type (for the whole group and the curative treatment subset only), performance status, chemotherapy use, and radiation dose (for the whole group and the SCC subgroup only); *p* ≤ 0.05 shown in bold.

**Table 3:** The final models for OS, LRFS, and MFS considering single SNPs, rs11730582 + rs187116, or rs11730582 + rs1126772 SNP combinations in the curative treatment and SCC subgroups.

Variable	HR (95% CI)	*p*
Curative Treatment Subgroup		
OS		
*OPN* rs11730582 CC Histology: SCC Ever smoking	1.66 (1.02–2.70) 2.09 (1.33–3.28) 3.11 (1.13–8.51)	0.042 0.001 0.027
LRFS		
*CD44* rs187116 GA/AA Histology: SCC Clinical stage III–IV	0.50 (0.29–0.88) 2.48 (1.40–4.40) 3.34 (1.03–10.84)	0.016 0.002 0.044
MFS		
*OPN* rs11730582 + *CD44* rs187116 combination: TC/CC + GG	2.05 (1.02–4.12)	0.043
SCC subgroup		
OS		
*OPN* rs1126772 AG/GG Ever smoking RT dose ≥ 60 Gy	1.54 (1.06–2.22) 3.42 (1.07–10.86) 0.50 (0.36–0.71)	0.022 0.037 7.7 × 10^−5^
*OPN* rs11730582 + rs1126772 combination: CC + AG/GG Ever smoking RT dose ≥ 60 Gy	2.85 (1.74–4.67) 3.53 (1.11–11.17) 0.50 (0.36–0.72)	3 × 10^−5^ 0.032 8.2 × 10^−5^

OS, overall survival; LRFS, locoregional recurrence-free survival; MFS, metastasis-free survival; HR, hazard ratio; CI, confidence interval; SCC, squamous cell carcinoma; RT, radiotherapy.

**Table 4:** Analysis for OS and MFS according to the *OPN* rs11730582–rs1126772 haplotypes in the curative treatment and SCC subgroups (haplotypes with *p* < 0.100 in multivariate models shown only).

SNP	Haplotype	Number of Copies	*p* Log-Rank	HR (95% CI)	*p*	HR (95% CI) ^a^	*p*
Curative treatment subgroup							
rs11730582–rs1126772	C-A	OS					
		0 1–2	0.227	1.0 1.28 (0.86–1.92)	0.229	1.0 1.81 (1.18–2.78)	**0.007**
		MFS					
		0 1–2	0.077	1.0 1.81 (0.93–3.54)	0.082	1.0 2.02 (0.99–4.13)	0.053
SCC subgroup							
rs11730582–rs1126772	T-A	OS					
		0 1–2	**0.026**	1.0 0.62 (0.43–0.91)	**0.014**	1.0 0.71 (0.47–1.06)	0.098
	C-G	OS					
		0 1–2	**0.033**	1.0 1.55 (1.08–2.24)	**0.019**	1.0 1.61 (1.10–2.38)	**0.016**

SNP, single nucleotide polymorphism; OS, overall survival; MFS, metastasis-free survival; HR, hazard ratio; CI, confidence interval. ^a^ Adjusted for age at diagnosis, sex, smoking, clinical stage, histology type (for the curative treatment subset only), performance status, chemotherapy use, and radiation dose (for the SCC subgroup only); *p* ≤ 0.05 shown in bold.

## Data Availability

The data presented in this study are available on reasonable request from the corresponding author.

## Supplementary Materials

The following supporting information can be downloaded at: https://www.mdpi.com/article/10.3390/cells12232721/s1; Figure S1: The haplotype block structure and linkage disequilibrium (LD) analysis for the polymorphisms examined in the study: (A) *OPN* rs4754, rs1126772, and rs11730582 and (B) *CD44* rs187116 and rs13347. The darkness of the cells indicates the strength of LD, and the numbers shown in the cells represent the pairwise *D*′ values expressed as percentages; Figure S2: The Kaplan–Meier plots according to the *OPN* haplotypes for (A) OS, (B) LRFS, and (C) MFS in the curative treatment subgroup. Only haplotypes with log-rank test *p* < 0.100 are shown; Figure S3: The Kaplan–Meier plots according to the *OPN* haplotypes for (A) LRFS and (B,C) OS in the SCC subgroup. Only haplotypes with log-rank test *p* < 0.100 are shown; Table S1: Genetic polymorphisms selected for the study and the genotype distribution; Table S2: Primers used in PCR-RFLP assay; Table S3: Osteopontin concentrations in plasma (ng/mL) according to the *OPN* genotypes.
