# Supplementary material for: Common Variants in Osteopontin and CD44 Genes as Predictors of Treatment Outcome in Radiotherapy and Chemoradiotherapy for Non-Small Cell Lung Cancer
Source: Cells. 2023 Nov 28;12(23):2721. doi: 10.3390/cells12232721 (PMC10706014; doi:10.3390/cells12232721)

Table S1. Genetic polymorphisms selected for the study and the genotype distribution.

SNP	Location GRCh38	Genotype	*n* (%) [a]	Region	Function class [b]	RegDB rank [c]	EUR MAF [d]	MAF	HWE *p* val
OPN rs1126772 A>G	chr4:87983034	AA<br>AG<br>GG	202 (66)<br>95 (31)<br>10 (3)	3′UTR	miRNA	1f	0.21	0.19	0.741
OPN rs11730582 T>C	chr4:87975269	TT<br>TC<br>CC	94 (31)<br>137 (45)<br>76 (24)	promoter	TFBS	1f	0.46	0.47	0.067
OPN rs4754 T>C Asp80Asp	chr4:87981540	TT<br>TC<br>CC	171 (56)<br>119 (39)<br>17 (5)	exon 6	S; splicing ESE/ESS	1f	0.28	0.25	0.529
CD44 rs187116 G>A	chr11:35144253	GG<br>GA<br>AA	105 (34)<br>144 (47)<br>58 (19)	intron 1	-	1f	0.46	0.42	0.475
CD44 rs13347 C>T	chr11:35231725	CC<br>CT<br>TT	195 (63)<br>104 (34)<br>8 (3)	3′UTR	miRNA	1b	0.22	0.20	0.175

SNP, single nucleotide polymorphism; MAF, minor allele frequency in the studied group; UTR, untranslated region; TFBS, transcription factor binding site; S, synonymous; ESE/ESS, exonic splicing enhancer/exonic splicing silencer; [a] Genotype frequency in the studied group; [b] According to https://snpinfo.niehs.nih.gov/snpinfo/snpfunc.html; [c] According to RegulomeDB v2.2, www.regulomedb.org; [d] MAF in European population according to www.ensembl.org.

Table S2. Primers used in PCR-RFLP assay.

Primer name	Primer sequence	PCR product length
OPN rs1126772 F	5′ GCATCTTCTGAGGTCAATTAAAAGG 3′	206 bp
OPN rs1126772 R	5′ CAGGGAGTTTCCATGAAGCCACAAACTAAACTAATTATCAAACACAC 3′	
OPN rs4754 F	5′ GATGATATGGATGATGAAGATGAAGA 3′	121 bp
OPN rs4754 R	5′AATGGTGAGACTCATCAGACTGG 3′	
CD44 rs187116 F	5′ AGGTGGTTGGAGATCACCTG 3′	153 bp
CD44 rs187116 R	5′ CTTTCGCAAGAACCACTTCC 3′	

F, forward primer; R, reverse primer.

Table S3. Osteopontin concentrations in plasma (ng/ml) according to the *OPN* genotypes.

SNP	Genotype	*n* (%)	OPN levels mean ± SD; median (range)	*p* value [a]
**All patients**				
OPN rs1126772 A>G	AA	202 (66)	116.6 ± 58.7; 103.6 (6.5–420.7)	0.808
	AG	95 (31)	126.1 ± 81.7; 103.5 (14.9–674.3)	
	GG	10 (3)	117.3 ± 39.2 ; 117.8 (52.1–183.5)	
OPN rs11730582 T>C	TT	94 (31)	125.4 ± 62.7; 110.3 (16.6–420.7)	0.125
	TC	137 (45)	119.6 ± 74.6; 99.7 (6.5–674.3)	
	CC	76 (24)	116.1 ± 53.0 ; 103.0 (44.8–255.7)	
OPN rs4754 T>C	TT	171 (56)	118.1 ± 59.9; 101.8 (6.5–420.7)	0.912
	TC	119 (39)	121.4 ± 72.4; 105.7 (44.8–674.3)	
	CC	17 (5)	121.0 ± 82.9; 96.6 (14.9–322.9)	
**Curative treatment subgroup**				
OPN rs1126772 A>G	AA	97 (67)	104.0 ± 49.4; 88.5 (6.5–327.6)	0.686
	AG	42 (29)	112.0 ± 102.7; 90.5 (14.9–674.3)	
	GG	6 (4)	100.1 ± 28.3; 102.8 (52.1–129.7)	
OPN rs11730582 T>C	TT	44 (30)	114.6 ± 57.2; 106.1 (16.6–327.6)	0.152
	TC	68 (47)	105.0 ± 81.6; 87.6 (6.5–674.3)	
	CC	33 (23)	109.4 ± 50.5; 93.2 (44.8–255.7)	
OPN rs4754 T>C	TT	79 (54)	105.7 ± 50.9; 88.5 (6.5–327.6)	0.829
	TC	57 (39)	111.5 ± 89.2; 91.9 (44.8–674.3)	
	CC	9 (7)	76.0 ± 43.8; 89.9 (14.9–129.7)	
**SCC subgroup**				
OPN rs1126772 A>G	AA	120 (66)	119.1 ± 59.8; 105.0 (6.5–342.5)	0.209
	AG	58 (32)	132.6 ± 93.5; 108.2 (14.9–674.3)	
	GG	3 (2)	152.5 ± 30.2; 152.5 (123.0–183.5)	
OPN rs11730582 T>C	TT	58 (32)	122.3 ± 58.9; 108.9 (31.5–327.6)	0.350
	TC	78 (43)	124.9 ± 89.7; 98.0 (6.5–674.3)	
	CC	45 (25)	124.5 ± 51.4; 112.0 (55.0–248.7)	
OPN rs4754 T>C	TT	105 (58)	119.1 ± 61.0; 103.2 (6.5–342.5)	0.508
	TC	70 (39)	129.1 ± 83.9; 109.1 (46.2–674.3)	
	CC	6 (3)	148.7 ± 102.9; 137.8 (14.9–322.9)	

SNP, single nucleotide polymorphism; OPN, osteopontin; SD, standard deviation; SCC, squamous cell carcinoma; [a] Kruskall-Wallis H test.

Figure S1. The haplotype block structure and linkage disequilibrium (LD) analysis for the polymorphisms examined in the study: (**A**) *OPN* rs4754, rs1126772 and rs11730582 and (**B**) *CD44* rs187116 and rs13347. The darkness of the cells indicates the strength of LD, and the numbers shown in the cells represent the pairwise *D′* values expressed as percentages.

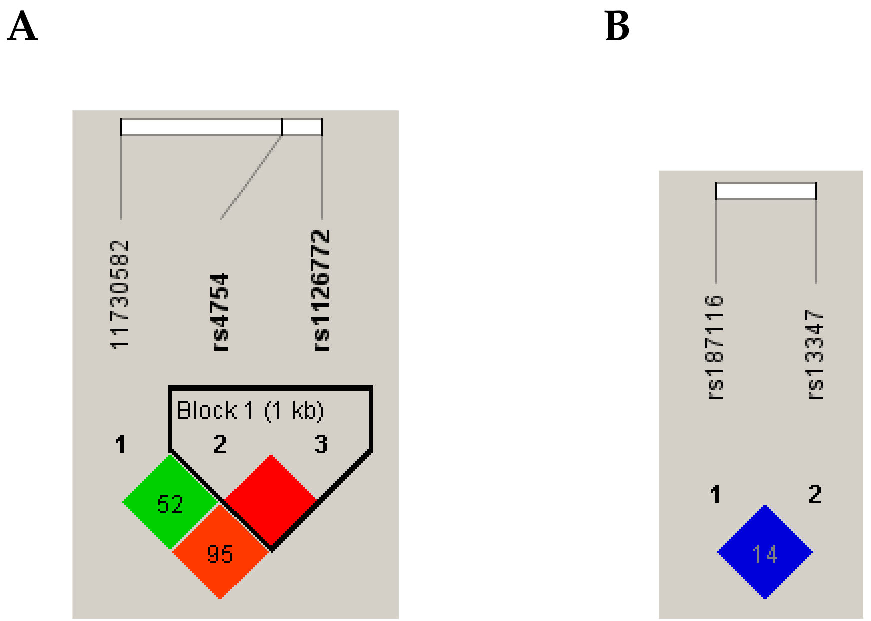

Figure S2. The Kaplan-Meier plots according to the *OPN* haplotypes for (**A**) OS, (**B**) LRFS and (**C**) MFS in the curative treatment subgroup. Only haplotypes with log-rank test $p <$ 0.100 are shown.

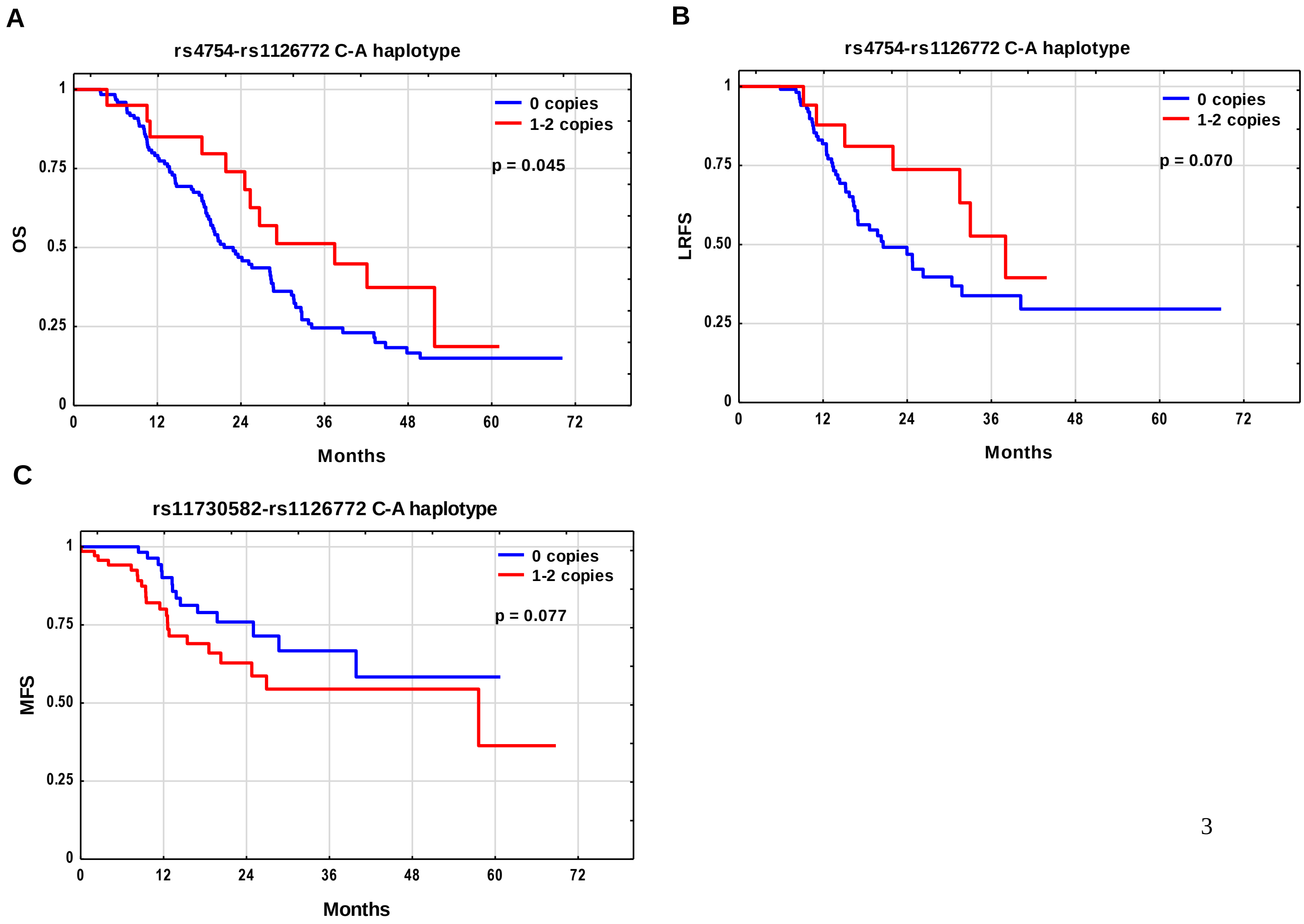

Figure S3. The Kaplan-Meier plots according to the *OPN* haplotypes for (**A**) LRFS and (**B, C**) OS in the SCC subgroup. Only haplotypes with log-rank test $p < 0.100$ are shown.

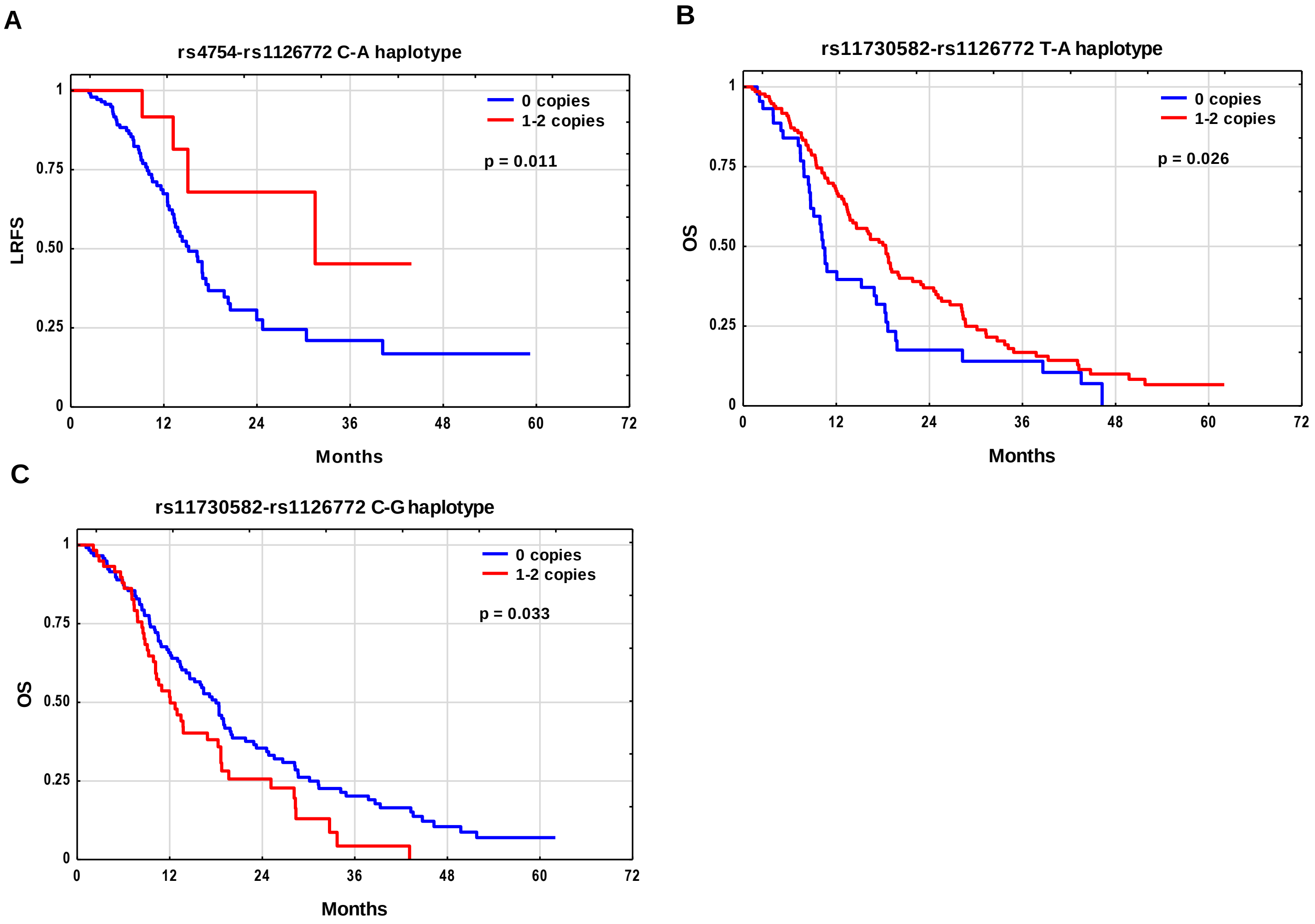

Supplement: Supplementary file 1 [file cells-12-02721-s001.zip › cells-2727601-supplementary.pdf]
